# CRISPR/Cas9 Knockout Strategies to Ablate *CCAT1* lncRNA Gene in Cancer Cells

**DOI:** 10.1186/s12575-018-0086-5

**Published:** 2018-11-01

**Authors:** Khadijeh Zare, Milad Shademan, Mohammad M. Ghahramani Seno, Hesam Dehghani

**Affiliations:** 1 Department of Basic Sciences, Faculty of Veterinary Medicine, Ferdowsi University of Mashhad, Azadi Square, Mashhad, Iran; 2 Division of Biotechnology, Faculty of Veterinary Medicine, Ferdowsi University of Mashhad, Azadi Square, Mashhad, Iran; 3 Stem Cell Biology and Regenerative Medicine Research Group, Research Institute of Biotechnology, Ferdowsi University of Mashhad, Azadi Square, Mashhad, Iran

**Keywords:** CRISPR/Cas9, Knockout, Cancer cell, Long noncoding RNA, CCAT1

## Abstract

**Background:**

With the increasing discovery of long noncoding RNAs (lncRNAs), the application of functional techniques that could have very specific, efficient, and robust effects and readouts is necessary. Here, we have applied and analyzed three gene knockout (KO) strategies to ablate the *CCAT1* gene in different colorectal adenocarcinoma cell lines. We refer to these strategies as “CRISPR excision”, “CRISPR HDR”, and “CRISPR du-HITI”.

**Results:**

In order to obstruct the transcription of lncRNA or to alter its structure, in these strategies either a significant segment of the gene is removed, or a transcription termination signal is inserted in the target gene. We use RT-qPCR, RNA-seq, MTT, and colony formation assay to confirm the functional effects of *CCAT1* gene ablation in knockout colorectal adenocarcinoma cell lines. We applied three different CRISPR/Cas9 mediated knockout strategies to abolish the transcription of CCAT1 lncRNA. CCAT1 knockout cells displayed dysregulation of genes involved in several biological processes, and a significant reduction for anchorage-independent growth. The du-HITI strategy introduced in this study removes a gene segment and inserts a reporter and a transcription termination signal in each of the two target alleles. The preparation of donor vector for this strategy is much easier than that in “CRISPR HDR”, and the selection of cells in this strategy is also much more practical than that in “CRISPR excision”. In addition, use of this technique in the first attempt of transfection, generates single cell knockouts for both alleles.

**Conclusions:**

The strategies applied and introduced in this study can be used for the generation of *CCAT1* knockout cell lines and in principle can be applied to the deletion of other lncRNAs for the study of their function.

**Electronic supplementary material:**

The online version of this article (10.1186/s12575-018-0086-5) contains supplementary material, which is available to authorized users.

## Background

Long non-coding RNAs (lncRNAs) play important roles in the regulation of transcription and post-transcriptional processes of coding and non-coding RNAs. Different mechanisms of function have been reported for lncRNAs including guiding chromatin modifiers to specific genomic loci, sequestering transcription factors, allosteric modulation of transcriptional regulatory proteins, alteration of nuclear domains, modulation of translation, modulation of mRNA stability, and working as competing endogenous RNAs [1–3]. Investigations on the role of lncRNAs in normal development and their aberrant functionality in different diseases underscore their importance in cellular phenomena such as genomic imprinting, dosage compensation, pluripotency, and differentiation commitment [4]. These findings have proven lncRNAs to be important molecules with significant functions.

Functional experiments to investigate the roles of lncRNAs are highly needed. A plethora of experiments has been devised and implemented to investigate the function of lncRNAs. These experiments can be divided into three categories. The first category includes RNA interference (RNAi) and antisense oligonucleotides that aim at the destruction of transcribed lncRNA. The second category includes clustered regularly interspaced short palindromic repeats (CRISPR)- mediated interference (CRISPRi) and activation (CRISPRa) that target the regulatory regions of the gene for tuning the level of its transcription. And the third category includes programmable nucleases including zinc finger nucleases (ZFNs), transcription activator-like effector nucleases (TALENs), and CRISPR/Cas9 (CRISPR-associated protein-9 nuclease) that are used for the ablation of the gene. Different criteria have been described for proper selection of a functional experiment for a given transcript [5]. These criteria are mainly related to the location of the target lncRNA gene and its position relative to other genes, the location of its regulatory regions [5], and sub-cellular localization of lncRNA. Knockdown of a lncRNA as a strategy to study its function has at least two limitations: (1) the incomplete depletion of the transcript due to the nature of the approach and the nuclear localization of lncRNA, and (2) possible off target effects [6, 7]. When transcription of the target gene is regulated in a complicated fashion, application of CRISPRi and CRISPRa techniques may also fail to inhibit or induce the lncRNA transcription [7]. For many lncRNAs with complex transcriptional profile and regulatory regions, the CRISPR/Cas9 mediated ablation of the locus can act as a very specific and powerful tool to study their functions.

The colon cancer associated transcript 1 (CCAT1) lncRNA is upregulated in various human malignant and pre-malignant tissues [8] including colon adenocarcinoma [9], gastric carcinoma [10–12], ovarian cancer [13], hepatocellular carcinoma [14], and other kinds of cancers. CCAT1 is known to be involved in various normal and pathologic cellular processes such as proliferation, migration and metastasis [15–18]; it was shown to also act as a competing endogenous RNA [19, 20], and a regulator of cMYC [15, 21]. However, due to the complexity of the processes that CCAT1 is known to control or contribute in, further investigations are required to clarify the exact molecular mechanism (s) by which this lncRNA acts. Gene knockdown procedures are helpful in delineating gene functions and RNAi [20–22] and antisense oligonucleotide [16] techniques have so far been used to study the CCAT1 functions. But, RNAi and antisense techniques act mostly within the cytoplasm, while many lncRNAs, including CCAT1, function mainly inside the nucleus and hence these techniques are far from being highly efficient [23].

In this work, we used three CRISPR/Cas9 mediated gene knockout strategies to deplete genomic CCAT1 transcription. The first strategy (herein named “CRISPR excision”) involved the complete removal (excision) of a DNA segment encompassing an exon. In the second strategy, a DNA segment coding for a DNA reporter construct including transcription terminator, was inserted in an exonic region in CCAT1 genomic locus by homology-directed repair (HDR) mechanism (herein named “CRISPR HDR”). And in the final strategy, hereafter called “CRISPR du-HITI” standing for “CRISPR/Cas9 dual allele homology-independent targeted integration”, we employed a modified version of CRISPR/Cas9 HITI [24]. In this strategy, in each allele, the targeting fragment replaces the genomic region located between two double-strand breaks (DSBs) formed by Cas9. In addition, two alleles are targeted simultaneously by two different inserts, making it possible to select the targeted genomic locus by both alleles. We successfully applied and exploited these three knockout strategies and established model cell lines lacking CCAT1 lncRNA.

## Results

### Application of Three Different CRISPR/Cas9 Mediated Knockout Strategies Targeting the CCAT1 lncRNA

In the human 8q24.21 gene desert region, the *CCAT1* gene (~ 11.8 Kb) is located ~ 173 kb downstream of the cancer susceptibility 21 (*CASC21*), and ~ 31 kb upstream of the cancer susceptibility 19 (*CASC19*) gene loci. CCAT1 transcription is highly upregulated in the pre-malignant adenomatous polyps and malignant colorectal carcinoma [8]. Two short and long isoforms of CCAT1 have been identified and it seems that the short isoform might be derived from the long isoform [25]. The long isoform of CCAT1 is totally retained in the nucleus. However, all the experiments designed to explore CCAT1 functions have so far used either RNAi or antisense oligonucleotides. To develop colorectal adenocarcinoma cellular models that lack CCAT1 transcription, we used three different strategies to knockout *CCAT1*. Our aim here was to interfere with the normal function of CCAT1 by removing a segment of the gene important for the secondary structure of CCAT1, or by introducing a transcription termination signal within the *CCAT1* locus to cause a premature transcription termination.

**Fig. 1:**
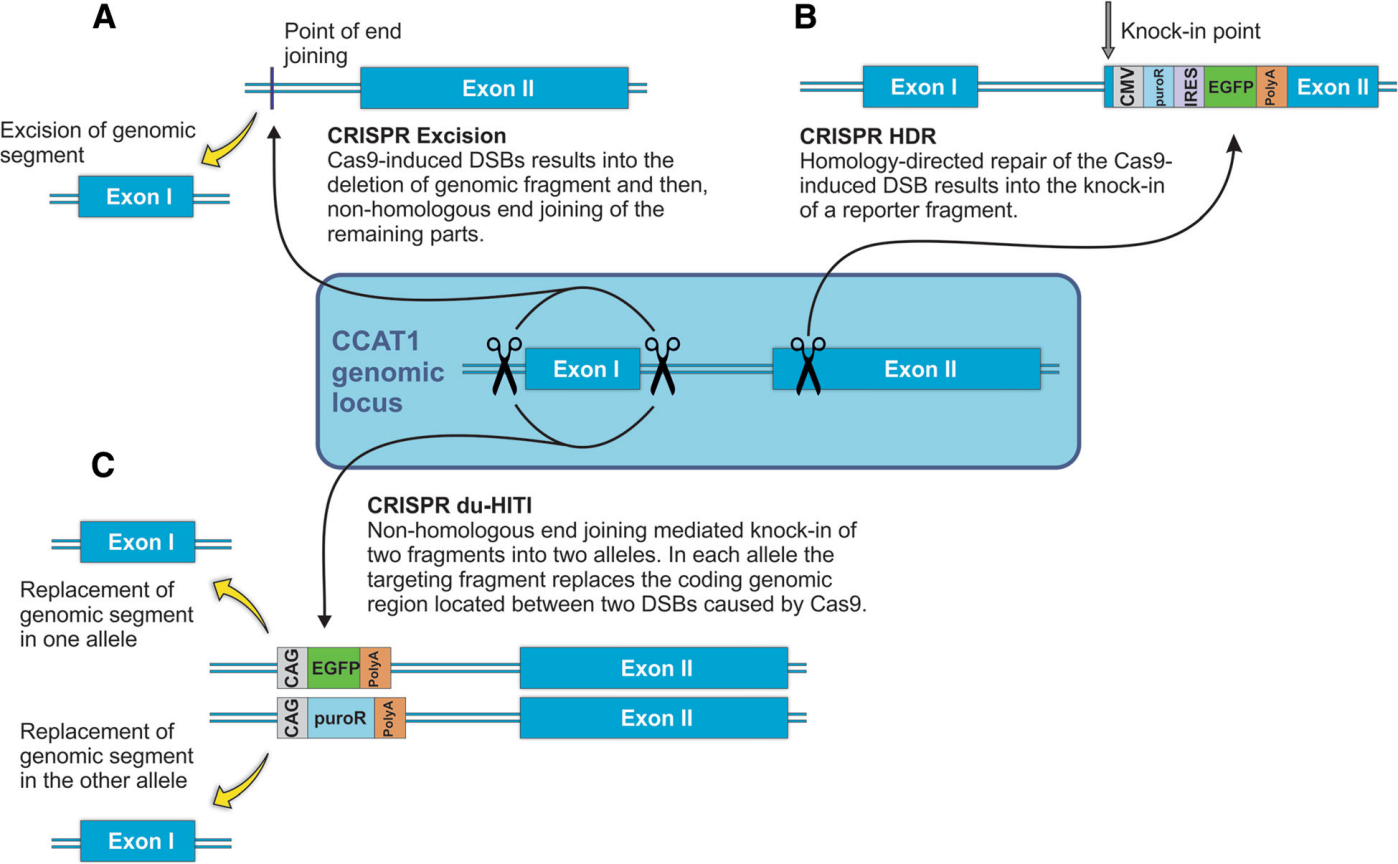
CRISPR/Cas9 knockout strategies for ablation of CCAT1 lncRNA gene. **a** “CRISPR excision”. To delete a genomic fragment (here, exon 1) two sgRNAs are targetted to either side of the fragment. Non- homologous end joining of the two remaining parts of genomic DNA after Cas9-induced double-strand breaks (DSBs) results in the deletion of the genomic fragment. **b** “CRISPR HDR”. In this strategy, using one sgRNA and Cas9-induced DSB in one region is followed by homology-directed repair using a reporter (CMV-PuroR-IRES2-EGFP) plus polyadenylation signal fragment (originated from a donor vector with homology arms). In this case, any transcript initiated from the first or second exon is confronted by a premature transcription termination. **c** “CRISPR du-HITI”. This strategy uses two donor vectors without homology arms. Two vectors containing sgRNA+PAM are used as donors, one with EGFP expression cassette, and the other with a PuroR expression cassette. Use of two sgRNAs directs the Cas9 protein towards the two either end of exon 1 at both alleles. Endonuclease function of Cas9 results into deletion of a genomic fragment (here, exon 1) from each allele, and linearization of two donor vectors. Selection of cells for their green color and their resistance to puromycin dihydrochloride results into cells with their both alleles targetted and knocked out

**Fig. 2:**
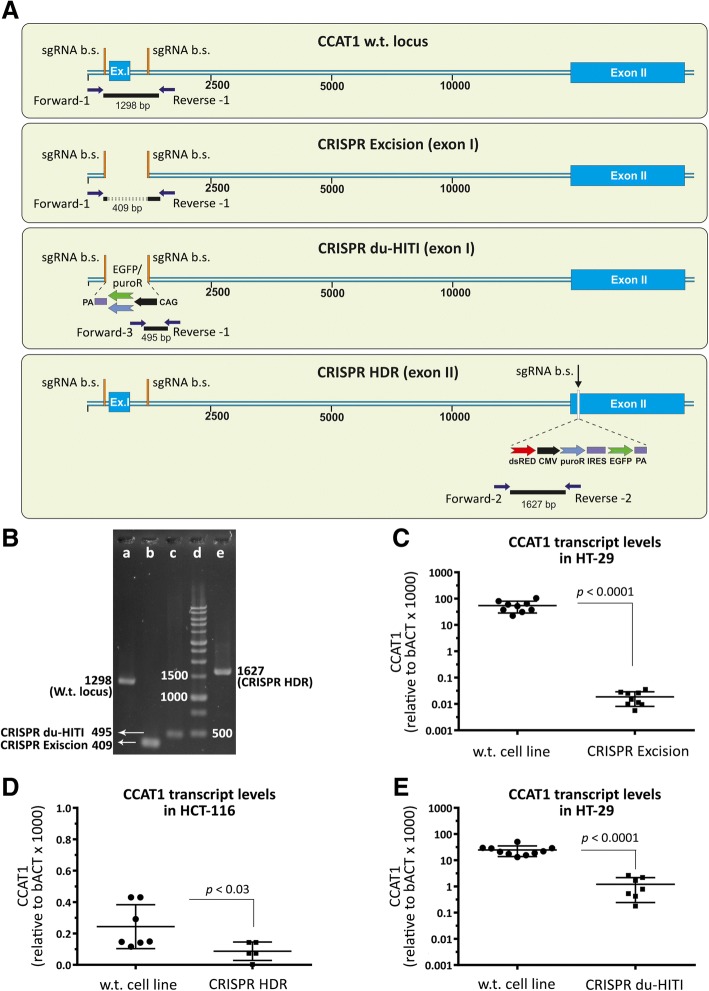
Confirmation of the *CCAT1* knockouts and qPCR analysis. **a** *CCAT1* wild-type locus and its knockout alleles by “CRISPR excision”, “CRISPR du-HITI”, and “CRISPR HDR”. Location of sgRNA binding sites (sgRNA b. s.), primers to perform PCR analysis (Table 4), and inserted fragments are shown. “CRISPR excision” of exon I (Ex. I) is confirmed by a 409 bp PCR fragment using two primers at either side of exon I. “CRISPR du-HITI” is confirmed by a PCR product of 495 bp using a primer in the insert and the other in the flanking region. The insert used for “CRISPR du-HITI” could express EGFP (bright green) or puroR (light blue) under CAG promoter. In this strategy when two alleles are targetted simultaneously, then the cells show both green fluorescence and resistance to puromycin dihydrochloride. “CRISPR HDR” was performed on exon II using an insert containing DSred (red in color), CMV, puroR, IRES, GFP, PA (poly adenylation signal, purple in color). In “CRISPR HDR” strategy cells with only one targetted allele could show both green fluorescence and resistance to puromycin dihydrochloride. PCR analysis using a primer in the insert and the other in the flanking region results in a 1627 bp product (Table 4). **b** Gel electrophoresis of PCR fragments for wild-type (w.t.) (**a**), CRISPR excised (b; clone # 20), “CRISPR du-HITI” knockout (c; clone # 27), and “CRISPR HDR” alleles (e; clone # 2i) according to the maps in panel A. Lane d is DNA size marker. **c** CCAT1 transcript relative to β-actin levels in wild-type and “CRISPR excision” knockout HT-29 colon adenocarcinoma cells. **d** CCAT1 transcript relative to β-actin levels in wild-type and “CRISPR HDR” knockout HCT-116 colon adenocarcinoma cells. **e** CCAT1 transcript relative to β-actin levels in wild-type and “CRISPR du-HITI” knockout SW-480 colon adenocarcinoma cells. In panels, C, D, and E the absolute copy number for CCAT1 and β-actin transcripts were quantified based on the related standard curves, and for three series of cell line cDNAs, the quantity of the CCAT1 transcript divided by the quantity of the β-actin was plotted. The statistical differences between the wild-type and knockout cell lines are analyzed by Mann-Whitney *U* test

The first strategy, that we here call “CRISPR excision”, involves precise deletion of a genomic fragment using two sgRNAs (Fig. 1a). In this strategy, we used two sgRNAs to direct the endonuclease activity of Cas9 to either side of CCAT1 exon 1 (Fig. 1a). For this purpose, we used HT-29, SW-480, and HCT-116 cell lines. After a first round of transfection and selection we obtained 45 HT-29 clones. PCR from genomic DNA revealed that 7 clones had one copy of CCAT1 deleted and no clones were homozygous for this deletion. We therefore used the heterozygous clones for a second round of “CRISPR excision” and after transfection and selection we were able to identify 2 out of 50 clones which were homozygous knockouts for CCAT1 as verified by PCR analysis of genomic DNA and sequencing of the PCR product (Additional file 1: Figure S1). RT-qPCR measurements of CCAT1 mRNA from the produced clones revealed a 370,000 fold (Fig. 2c) reduction of CCAT1 mRNA in the knockout clones compared to the wild-type cells. Previous reports achieved just a ~ 10 fold knockdown of CCAT1 in HT-29 cells using antisense oligonucleotides [25].

The second strategy that we here refer to as “CRISPR HDR” involved the insertion of a reporter gene and a transcription termination signal in CCAT1 genomic locus using a single sgRNA and a donor vector with homology arms (Fig. 1b). We targeted the initial regions of the second exon since it has been reported that this region acts as a promoter for transcripts that originate from exon 2. The inclusion of a transcription termination signal in exon 2 ensures that the transcription of transcripts originating from exon 1 or from the proximal region of exon 2 will cease prematurely. Our reporter construct includes a green fluorescence protein (GFP) gene and DNA for the resistance to puromycin dihydrochloride to facilitate selection of correctly modified cells. In the first attempt of selection of puromycin resistant and green colonies, we selected 6 SW-480 and 14 HCT-116 clones. The selected clones were clonally expanded and were subjected to DNA extraction, PCR validation, and Sanger sequencing (Additional file 1: Figure S1). These analyses indicated that 4 out of 6 selected SW-480 and 8 out of 14 selected HCT-116 clones harbored the desired fragment insertions. RT-qPCR analysis of these clones revealed statistically significant lower expression levels (2 fold reduction) of CCAT1 compared to the control cells (Fig. 2d).

Our third strategy, here referred as “CRISPR du-HITI” comprises the deletion of a genomic fragment encompassing exon I of CCAT1 using two sgRNAs and insertion of two reporters and transcription termination signals using two donor vectors without homology arms (Fig. 1c). Cloning of the donor vectors used for this strategies was less laborious than that used for “CRISPR HDR” and the presence of a selection made the screening of correctly modified cells more practical compared to the “CRISPR excision” approach. In addition, using this strategy it is possible to mostly identify homozygous deletion of the target gene by virtue of selection for both GFP and puromycin. In the selected clones one allele will be therefore have the GFP reporter and the other allele the PuroR cassette. Furthermore, in this strategy, it is also possible to delete large genomic fragments and replace them with different reporter cassettes. In the first attempt of transfection, single cell isolation, and clonal expansion, we were able to acquire 6 clones of HCT-116 cells that had permanent green fluorescence phenotype and puromycin resistance (Fig. 2). The knock-in was verified by PCR analysis that showed the insertion of the desired fragments (Additional file 1: Figure S1). These clones had statistically significant lower expression levels (27 fold reduction) of CCAT1 compared to the control cell line (Fig. 2e).

### *CCAT1* Knockout Cells Display Dysregulation of Genes Involved in Several Biological Processes

We used RNA-Seq to evaluate changes in the transcriptional landscape associated with inactivation of CCAT1 gene in colon adenocarcinoma cells (CCAT1 KO cells). We tested for enriched GO (Gene Ontology) terms in the wild-type versus knockout transcriptomes. Differentially expressed (DE) transcripts (log2-fold change > 1 and < − 1), statistically significant (*p* < 0.05) for enrichment in the CCAT1 KO cells, were identified. GO analysis indicated that a total of 332 genes were associated with some biological processes, molecular functions, or cellular components. The enriched GO terms in the category of molecular function, biological process, and cellular components are shown in Tables 1, 2 and 3.

**Table 1:** Enriched gene ontology terms in the CCAT1 wild-type versus CCAT1 knockout transcriptomes derived from differentially expressed genes (in the category of molecular function)

ID	Term	Gene Count	*p* Value^a^
GO:0004867	serine-type endopeptidase inhibitor activity	9	2.50E-04
GO:0016491	oxidoreductase activity	12	6.60E-04
GO:0005515	protein binding	171	9.60E-03
GO:0008236	serine-type peptidase activity	5	2.30E-02
GO:0004879	RNA polymerase II transcription factor activity, ligand-activated sequence-specific DNA binding	4	2.30E-02
GO:0004252	serine-type endopeptidase activity	10	3.10E-02
GO:0015485	cholesterol binding	4	3.30E-02
GO:0004029	aldehyde dehydrogenase (NAD) activity	3	3.30E-02
GO:0032810	sterol response element binding	2	3.40E-02
GO:0003727	single-stranded RNA binding	4	3.70E-02
GO:0016620	oxidoreductase activity, acting on the aldehyde or oxo group of donors, NAD or NADP as acceptor	3	4.10E-02
GO:0004715	non-membrane spanning protein tyrosine kinase activity	4	4.40E-02
GO:0005506	iron ion binding	7	4.70E-02
GO:0003779	actin binding	10	4.90E-02

^a^Only GO terms with *p* value of less than 0.05 are shown

**Table 2:** Enriched gene ontology terms in the CCAT1 wild-type versus CCAT1 knockout transcriptomes derived from differentially expressed genes (in the category of biological process)

ID	Term	Gene Count	*p* Value^a^
GO:0042493	response to drug	17	4.50E-05
GO:0055114	oxidation-reduction process	24	1.30E-04
GO:0031295	T cell costimulation	7	1.80E-03
GO:0008283	cell proliferation	15	3.00E-03
GO:0030522	intracellular receptor signaling pathway	5	3.40E-03
GO:0010951	negative regulation of endopeptidase activity	8	3.90E-03
GO:0090090	negative regulation of canonical Wnt signaling pathway	9	5.60E-03
GO:0008285	negative regulation of cell proliferation	15	6.00E-03
GO:0006805	xenobiotic metabolic process	6	9.20E-03
GO:0051897	positive regulation of protein kinase B signaling	6	1.20E-02
GO:0030449	regulation of complement activation	4	1.30E-02
GO:0006081	cellular aldehyde metabolic process	3	1.30E-02
GO:0048013	ephrin receptor signaling pathway	6	1.40E-02
GO:0001822	kidney development	6	1.40E-02
GO:0007612	learning	5	1.40E-02
GO:0032091	negative regulation of protein binding	5	1.40E-02
GO:0046685	response to arsenic-containing substance	3	1.90E-02
GO:0007568	aging	8	1.90E-02
GO:0060337	type I interferon signaling pathway	5	2.10E-02
GO:0042632	cholesterol homeostasis	5	2.10E-02
GO:0043524	negative regulation of neuron apoptotic process	7	2.20E-02
GO:0006954	inflammatory response	13	2.30E-02
GO:0016477	cell migration	8	2.40E-02
GO:0006636	unsaturated fatty acid biosynthetic process	3	2.50E-02
GO:0032570	response to progesterone	4	2.60E-02
GO:0038083	peptidyl-tyrosine autophosphorylation	4	2.80E-02
GO:0008360	regulation of cell shape	7	2.80E-02
GO:0032526	response to retinoic acid	4	3.00E-02
GO:0002223	stimulatory C-type lectin receptor signaling pathway	6	3.00E-02
GO:0003057	regulation of the force of heart contraction by chemical signal	2	3.30E-02
GO:0045785	positive regulation of cell adhesion	4	3.30E-02
GO:0042127	regulation of cell proliferation	8	3.40E-02
GO:0031668	cellular response to extracellular stimulus	3	3.50E-02
GO:0009615	response to virus	6	3.50E-02
GO:0030325	adrenal gland development	3	3.80E-02
GO:0071222	cellular response to lipopolysaccharide	6	3.90E-02
GO:0030154	cell differentiation	14	4.30E-02
GO:0014070	response to organic cyclic compound	4	4.70E-02
GO:0070668	positive regulation of mast cell proliferation	2	4.90E-02
GO:0060675	ureteric bud morphogenesis	2	4.90E-02
GO:0006991	response to sterol depletion	2	4.90E-02

^a^Only GO terms with *p* value of less than 0.05 are shown

**Table 3:** Enriched gene ontology terms in the CCAT1 wild-type versus CCAT1 knockout transcriptomes derived from differentially expressed genes (in the category of cellular component)

ID	Term	Gene Count	*p* Value^a^
GO:0070062	extracellular exosome	78	4.50E-06
GO:0005615	extracellular space	42	1.30E-04
GO:0005829	cytosol	78	1.10E-03
GO:0005576	extracellular region	42	4.20E-03
GO:0031234	extrinsic component of cytoplasmic side of plasma membrane	6	5.50E-03
GO:0002102	podosome	4	7.20E-03
GO:0005856	cytoskeleton	14	9.80E-03
GO:0030687	preribosome, large subunit precursor	4	1.20E-02
GO:0031528	microvillus membrane	3	3.90E-02
GO:0005886	plasma membrane	83	4.10E-02
GO:0072562	blood microparticle	7	4.20E-02
GO:0034666	integrin alpha2-beta1 complex	2	4.90E-02

^a^Only GO terms with *p* value of less than 0.05 are shown

### *CCAT1* Knockout Cells Show Reduced Anchorage-Independent Growth

**Fig. 3:**
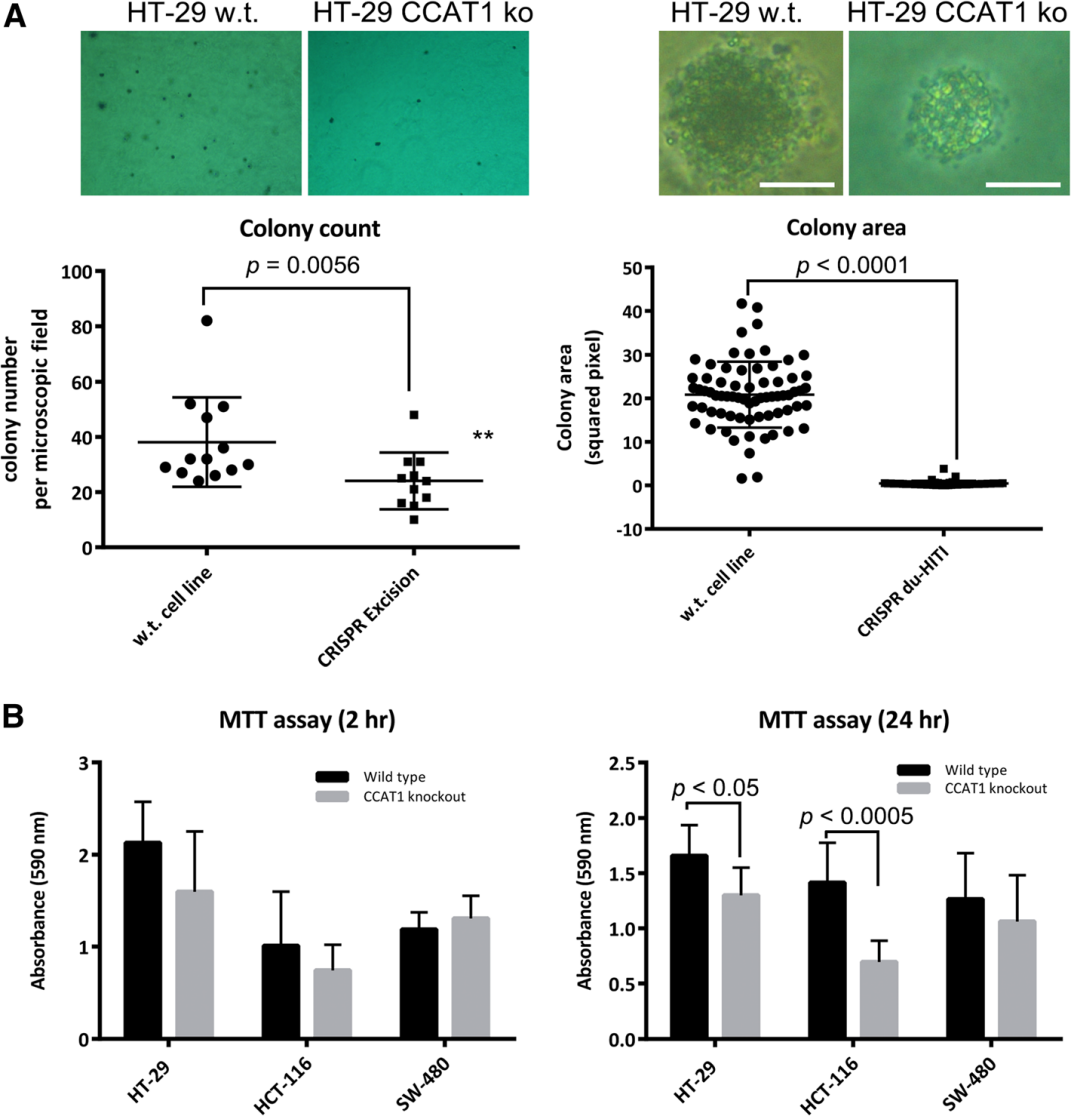
*CCAT1* knockout cells lose the capacity for anchorage-independent growth. **a** Representative images of soft agar colony formation assay for wild-type and *CCAT1* KO cell lines and evaluation of colony count and colony area in these lines. **b** MTT assay in HT-29, HCT-116, and SW-480 wild-type and *CCAT1* KO cell lines performed 2 h and 24 h after seeding. The statistical differences between the wild-type and knockout cell lines are analyzed by Mann-Whitney test. The scale bars in panel A denote 200 μm

The role of CCAT1 in anchorage-independent growth was analyzed by soft agar colony formation assay. It is known that inhibition of entosis enhances anchorage-independent growth in soft agar assay and promotes tumorigenesis [26]. HT-29, HCT-116, and SW-480 wild-type and *CCAT1* KO cells were cultured in soft agar and allowed to form colonies over a period of 3 weeks. The wild-type HT-29 cells formed an average of 35 ± 4 colonies per microscopic fields, while *CCAT1* KO HT-29 cells formed an average of 24 ± 3 colonies. The average colony size of wild-type SW-480 colonies was 20.86 ± 0.9, while the average size of *CCAT1* KO SW-480 cells was 0.4 ± 0.08 pixel squared (Fig. 3a).

MTT assay was performed to investigate whether the decrease in the number and size of soft agar colonies is associated with changes in the metabolic activity of *CCAT1* KO cells. MTT assay performed 2 h after cell seeding did not reveal any significant changes between KO and wild-type cells. However, MTT assay after 24 h exhibited a significant decrease in the absorbance readings in two *CCAT1* KO cell lines compared to the wild-type cells (Fig. 3b). This suggested that the decreased MTT absorbance reading after 24 h might be caused by the lower number of cells in the knockout groups, while this effect is not evident when cells are cultured only for 2 h and have not started to divide [27, 28].

## Discussion

With the increasing discovery of lncRNAs, there is a growing need for application of functional studies to reveal their biological roles. Similar to protein-coding genes, approaches aiming at expression inhibition are useful for studying lncRNA biological functions. However, the two approaches routinely exploited for this purpose, i.e. RNA interference and antisense strategies, are not as efficient for lncRNAs with nuclear localization. Furthermore, these techniques are associated with poor depletion of the target gene, off-target effects, and technical variations. Applying newly developed genome editing approaches to alter lncRNA genomic locus so that the related lncRNA becomes non-functional is a good alternative. However, considering the non-coding nature of lncRNAs, changes at the corresponding genomic loci need to be large enough to adversely affect the structure and biological function of the expressed RNA. For example, a simple excision followed by NHEJ may not provide enough structural changes in a given lncRNA. Here, we used three different CRISPR-mediated KO strategies, i.e. “CRISPR excision”, “CRISPR HDR”, and “CRISPR du-HITI”, to alter the *CCAT1* locus in different colorectal adenocarcinoma cell lines.

“CRISPR excision” using two sgRNAs to delete a genomic fragment of lncRNA genes has already been reported [29–31]. For the implementation of this strategy, no donor vectors are used and thus, no reporter genes are inserted into the genome. As a result, the selection of KO cells becomes a tedious job. “CRISPR HDR”, in contrast, uses donor vectors and thus, enables the user to select the targeted cells based on fluorescence and/or resistance to an antibiotic [30, 31]. However, in this strategy, homology arms need to be cloned into the donor vector. The “CRISPR du-HITI” which doesn’t rely on the presence of homology arms, makes the selection of the target cells easier (Fig. 1). This approach enables us to direct fabrication of KOs so that each cell with both antibiotic resistance and fluorescence phenotypes is easily selected as a dual allele KO.

*CCAT1* KO cells created in this study were verified by genomic PCR, sequencing, and RT-qPCR (Fig. 2). Since the qPCR forward and reverse primers were complementary to exon 1 and exon 2, respectively, we did not expect to have any amplification products from “CRISPR excision” and “CRISPR du-HITI” KO cells. In these two approaches, exon 1 was completely removed. However, in “CRISPR HDR” the first segment of exon 2 (after the complementary sequence to reverse primer) was targeted with a fragment containing reporter/transcription termination signal. Thus, an amplification product was expected. This could explain the presence of RNA which was still measurable in these cells, and that only the two-fold CCAT1 decrease was observed (Fig. 2d). These three cell lines express different levels of CCAT1, the highest level of CCAT1 is detected in HT-29, while both SW-480 and HCT 116 express low levels of CCAT1 (Additional file 2: Figure S2). Presence of a residual RNA (the cycle threshold for knockout CCAT1 in all cell lines) could also be attributed to the cancerous nature of these cells and the presence of CCAT1 extra copies and pseudogenes in the genome.

The *CCAT1* KO cells were subjected to RNA-seq, soft agar colony formation assay, and MTT analysis. These cells displayed differential expression of a number of genes (Tables 1-3). Accordingly, soft agar colony formation and MTT assays were able to verify that cell proliferation is negatively affected in the *CCAT1* KO cells (Fig. 3). This finding in turn, is in accordance with the previous reports that CCAT1 suppression affects genes that are involved in the regulation of cell proliferation [32].

## Conclusions

In conclusion, we characterized here three CRISPR/Cas9 mediated strategies that provide effective tools for lncRNA functional surveys. Furthermore, the *CCAT1* KO cell lines created in this study can be used for further functional analyses to reveal the battery of functions of CCAT1. The du-HITI strategy introduced in this study is easy to implement and can be applied for the generation of homozygous cells knockout for a specific lncRNA of interest.

## Methods

### Cell Culture

All cell lines were purchased from the National Cell Bank of Iran (Pasteur Institute, Iran) and cultured as recommended by the ATCC. Three colorectal adenocarcinoma cells were used, which differed based on microsatellite stability (MS) and CpG island methylator phenotype (CIMP). HT-29 cells are microsatellite stable (MSS), and CIMP high, SW-480 cells are MSS and CIMP low, and HCT-116 cells show microsatellite instability (MSI) and are CIMP high [33]. HT-29 and SW-480 cells were cultured in RPMI (Biowest, France) supplemented with 10% FBS (Gibco, USA), 50 U/ml penicillin and 50 μg/ml streptomycin (Sigma-Aldrich, USA) at 37 °C in 5% CO2. HCT-116 cells were cultured in DMEM (Gibco, USA) containing 10% FBS and penicillin-streptomycin (50/50 U/ug/ml) at 37 °C in a 5% CO2 environment.

### DNA Constructs and Gene Targeting

The single guide RNA (sgRNA) sequences targeting different segments of *CCAT1* gene were designed using the CRISPR design tool (http://crispr.mit.edu/). Three vectors of pX459 (containing U6 promoter-sgRNA insertion site-sgRNA scaffold, and CAG promoter-Cas9-T2A- puromycin N-acetyltransferase gene-bovine growth hormone polyadenylation signal), pX460–1 (containing U6 promoter-sgRNA insertion site-sgRNA scaffold, and CAG promoter-enhanced GFP (EGFP)-bovine growth hormone polyadenylation signal), and pX461–1 (containing U6 promoter-sgRNA insertion site-sgRNA scaffold, and CAG promoter-puromycin N-acetyltransferase (PuroR)-bovine growth hormone polyadenylation signal) were used for sub-cloning of sgRNAs. For this purpose, oligonucleotides (Table 4) containing the sgRNA expressing sequence and BbsI steaky ends were synthesized (Macrogen Inc., South Korea), annealed, phosphorylated and ligated into the BbsI-digested and gel purified (using Gel Extraction Kit; DENAzist Asia Co., Iran) vectors. For “CRISPR du-HITI” targeting vectors, the PAM sequence was also introduced after the sgRNA expressing sequence.

**Table 4:** Oligonucleotides used in this study

Gene	Sequence (5′ to 3′)	Product (bP)	Application
CCAT1 (NR_108049.1)	F: CTGACAACATCGACTTTG R: CTCACAGTTTTCAAGGGA Probe: FAM-CTTAGCCATACAGAGCCAACCTG-BHQ1	108	qPCR
	F: CGATCGttctgttttcaatggggatt R: TCGAGggagctgcggataacagcatat	546	Cloning of left homology arm
	F: CTAGTCCCgcatcacagctactgtcaaccc R: ATCCCCtcaaagcacttctgtggtagga	832	Cloning of right homology arm
	Forward-1: CACATGGCTCCCATCACACTA Reverse-1: GGGGGAAGAAATTTAAGATGCACA	1298 (w.t.) 409 (ko)	PCR confirmation of CRISPR Excision knockout allele
	Forward-2: CACGCAGATCACATGACCCT Reverse-2: CGGGCCATTTACCGTAAG	1627 (ko)	PCR confirmation of CRISPR HDR knockout allele
	Forward-2: CACGCAGATCACATGACCCT R: AAACgatggagctgcggataacagC	843 (w.t.)	PCR confirmation of CRISPR HDR wild-type allele
	Forward-3: CGGGCCATTTACCGTAAG Reverse-1: GGGGGAAGAAATTTAAGATGCACA	495 (ko)	PCR confirmation of CRISPR du-HITI knockout allele
	F: CACCGaatcggagtccaaagccatt R: AAACaatggctttggactccgattC	–	sgRNA (downstream of Exon 1)
	F: CACCGataatggaggggatttacgt R: AAACacgtaaatcccctccattatC	–	sgRNA (upstream of Exon 1)
	F: CACCGctgttatccgcagctccatc R: AAACgatggagctgcggataacagC	–	sgRNA (Exon 2)
	F: CACCGaatggctttggactccgatttgg R: AAACccaaatcggagtccaaagccattC	–	sgRNA bait (+PAM) (downstream of Exon 1)
b-Actin	F: TGCAGAAGGAGATCACTG R: CTTGCTGATCCACATCTG Probe: CY5-AAGATCAAGATCATTGCTCCTCCTGA-BHQ2	141	qPCR

The vector used for “CRISPR HDR” targeting contained left homology arm (546 bp), DsRed2, herpes simplex virus thymidine kinase polyadenylation signal, CMV promoter, PuroR, IRES2, EGFP, SV40 polyadenylation signal, and right homology arm (832 bp).

According to Table 5, constructs (verified by Sanger sequencing; Macrogen Inc., South Korea) were used for transfection of colorectal adenocarcinoma cell lines using Lipofectamine 2000 reagent (Thermo Fisher Scientific, USA), Exgen 500 (Thermo Fisher Scientific, USA) or Polyethylenimine (Sigma-Aldrich, USA). For “CRISPR excision” and removal of *CCAT1* exon 1, 2 weeks after transfection cells were cultured in low density, and individual colonies (50 colonies) were allowed to expand and then selected by PCR analysis. Selection of colonies for “CRISPR du-HITI” and “CRISPR HDR” was performed by their GFP expression and resistance to puromycin dihydrochloride (Sigma-Aldrich, USA). All “CRISPR excision”, “CRISPR du-HITI”, and “CRISPR HDR” individual colonies were verified by genomic DNA isolation, PCR analysis, and Sanger sequencing (Macrogen Inc., South Korea).

**Table 5:** DNA constructs used in this study

Construct	Features	Application
pX459_2	hU6 promoter- sgRNA (downstream of CCAT1 Exon 1)-sgRNA scaffold-CAG promoter-Cas9-T2A- PuroR-bGH polyA	CRISPR Excision CRISPR du-HITI
pX459_3	hU6 promoter- sgRNA (upstream of CCAT1 Exon 1)-sgRNA scaffold-CAG promoter-Cas9-T2A- PuroR-bGH polyA	CRISPR Excision CRISPR du-HITI
pX459_13	hU6 promoter- sgRNA (CCAT1 Exon 2)-sgRNA scaffold-CAG promoter-Cas9-T2A- PuroR-bGH polyA	CRISPR HDR
pHD_4317_CCAT1 E2 HAs	Exon 2 LHA-DsRed2-HSV TK polyA-CMV promoter –PuroR-IRES2-EGFP-SV40 polyA-Exon 2 RHA	CRISPR HDR
pX460_11	hU6 promoter-sgRNA (downstream of CCAT1 Exon 1) plus PAM-sgRNA scaffold-CAG promoter-EGFP-bGH polyA	CRISPR du-HITI
pX461_11	hU6 promoter-sgRNA (downstream of CCAT1 Exon 1) plus PAM-sgRNA scaffold-CAG promoter-PuroR-bGH polyA	CRISPR du-HITI

*hU6* human U6 promoter, *sgRNA* single guide RNA, *PuroR* puromycin N-acetyltransferase, *bGH* bovine growth hormone polyadenylation signal, *SV40 polyA* SV40 polyadenylation signal, *IRES* internal ribosome entry site

### Genomic DNA Isolation and Analysis

Genomic DNA was isolated with the Genomic DNA Isolation Kit I (DENAzist Asia Co., Iran) from wild-type and knockout cell lines and was subjected to PCR amplification (Fig. 2). PCR-amplified bands after clean-up and reaction recovery (DENAzist Asia Co., Iran), were subjected to Sanger sequencing (Macrogen Inc., South Korea).

### Reverse Transcription Quantitative PCR

Total RNA was isolated from wild-type and knockout cell lines using Total RNA Isolation Kit (DENAzist Asia Co., Iran). The quality and quantity of extracted RNA were evaluated using gel electrophoresis and a 2000 Nanodrop spectrophotometer (Thermo Scientific, USA). Total RNA (1 μg) was reverse transcribed using random hexamer primers and MMLV reverse transcriptase (Thermo Fisher Scientific, USA). To quantify the level of transcripts for CCAT1 and β-actin, quantitative RT-PCR reactions containing Premix Ex Taq (Probe qPCR) master mix (Takara, Japan), 2 μl cDNA template and each primer at 500 nM and 100 nM probe (dual-labeled hybridization probes, 5’FAM-3’BHQ1-labeled for CCAT1 and 5’CY5–3’BHQ2 for β-actin) in a 20 μl reaction volume, were carried out in a Rotor-Gene Q real-time PCR cycler (Qiagen, USA). Amplification steps were: 95 °C for 5 min, followed by 40 cycles of 94 °C for 30 s, 57.5 °C for 30 s, and 72 °C for 30 s. To confirm the identity of PCR products, Sanger sequencing was performed (Macrogen Inc., South Korea).

Amplified fragments were sub-cloned in pTZ57R or pGATA plasmids and their serial dilution was used to make standard curves. Each dilution was subjected to three PCR reactions and real-time readings were performed in triplicate. Then, the log of copy numbers was plotted against cycle threshold (Ct) numbers. For each qPCR reaction, efficiency (E) was calculated from the calculated slope of standard curves, generated using 5 fold serially diluted solutions of plasmids, according to the following equation: E = (10^–1/slope-1^) × 100%. All standard curves were linear in the analyzed range with an acceptable correlation coefficient (R^2^). Absolute copy number for CCAT1 and β-actin transcripts were quantified based on the related standard curves. For three series of cDNAs, the quantity of the target transcript (CCAT1) was divided by the quantity of the reference gene (β-actin) and plotted.

### RNA Sequencing and GO Analysis

Tuxedo pipeline [34] was performed for differential expression analysis of whole transcriptome sequencing results of HT-29 wild-type and knockout cell lines. The differentially expressed genes from the study were input for assessing the enrichment. For data precision and consistency, we adjusted *p*-value to correct the data, fold change was declared at least ±1 fold and *p*-value < 0.05 was regarded as significant. Compared to the wild-type cell lines, 182 differentially expressed genes were selected and association of gene list to Gene Ontology (GO) terms was performed using the new GO category (GO Direct) of the latest released version of DAVID web tool (DAVID 6.8 Oct. 2016), (http://david.ncifcrf.gov) [35, 36].

### Soft Agar Colony Formation and MTT Assays

The methodology previously described was followed [37]. To evaluate the anchorage-independent growth of *CCAT1* KO cell lines, cells were plated at 2 × 10^4^ in DMEM/F12 containing 5% horse serum, penicillin/streptomycin, and 0.3% 2-hydroxyethyl agarose (Sigma, USA) (with 0.6% 2-hydroxyethyl agarose underlay) in flat-bottom non-treated six-well plates (SPL, South Korea). By overlaying 1 ml of 0.3% 2-hydroxyethyl agarose/medium solution onto the existing feeder layer, the feeding procedure was performed for three times (once a week). After 3 weeks, the colonies were fixed with 4% paraformaldehyde (Electron Microscopy Sciences, USA) in PBS for 30 min and stained with 0.5% methylene blue in ethanol for 30 min. The number and area of colonies were determined using NIH ImageJ software [38].

To perform MTT (3-(4,5-Dimethylthiazol-2-Yl)-2,5-Diphenyltetrazolium Bromide) assay, Cells were seeded in a 96-well plate in three or more replicates (5000 cells/well). and MTT (to a final concentration of 0.5 mg/ml in culture medium) was added to each well after 2 or 24 h of seeding. The plate was incubated at 37 °C in dark for 3 h. Then, the media was discarded, 100 μl of DMSO was added to each well, incubated at room temperature in the dark for 15 min, and the color developed was measured at 590 nm using Epoch 2 microplate reader (BioTek Instruments Inc., USA).

## Additional files

Additional file 1: **Figure S1.** PCR validation of CCAT1 knockout cells. PCR amplification of wild-type (w.t.) and knockout (KO) alleles in different clones (different numbers) produce by CRISPR excision (A), CRISPR HDR (B), and CRISPR du-HITI (C) approaches are shown. Colonies 18, 19, and 20 in panel A are CCAT1 wild-type, heterozygous CCAT1 KO, and homozygous CCAT1 KO, respectively. In panel B, colonies 8e and 2i are heterozygous CCAT1 KO. In panel C, colony n5 is a homozygous CCAT1 KO. The primers used for PCR amplification are listed in Table 4. M: DNA size marker, bp: base pair. (TIF 3887 kb)

Additional file 2: **Figure S2.** Amplification curves for CCAT1 transcript in HT29, SW480, and HCT116 cells. The representative cycle thresholds (CTs) are shown for each cell line. The big difference in CT values indicates that the level of CCAT1 transcript in HT29 is much higher than that in SW480 and HCT116 cells. NTC: non-template control. (TIF 944 kb)

## Abbreviations

CCAT1: Colon cancer associated transcript 1
lncRNA: Long noncoding RNA
KO: Knockout
CRISPR: Clustered regularly interspaced short palindromic repeats
Cas9: CRISPR-associated protein-9 nuclease
HDR: Homology-directed repair
HITI: Homology-independent targeted integration
du-HITI: Dual allele HITI
RNAi: RNA interference
DSBs: Double-strand breaks
DE: Differentially expressed
RT: Reverse transcription
qPCR: Quantitative PCR
