# Supplementary figures and images for: CRISPR/Cas9 Knockout Strategies to Ablate CCAT1 lncRNA Gene in Cancer Cells
Source: Biol Proced Online. 2018 Nov 1;20:21. doi: 10.1186/s12575-018-0086-5 (PMC6211572; doi:10.1186/s12575-018-0086-5)

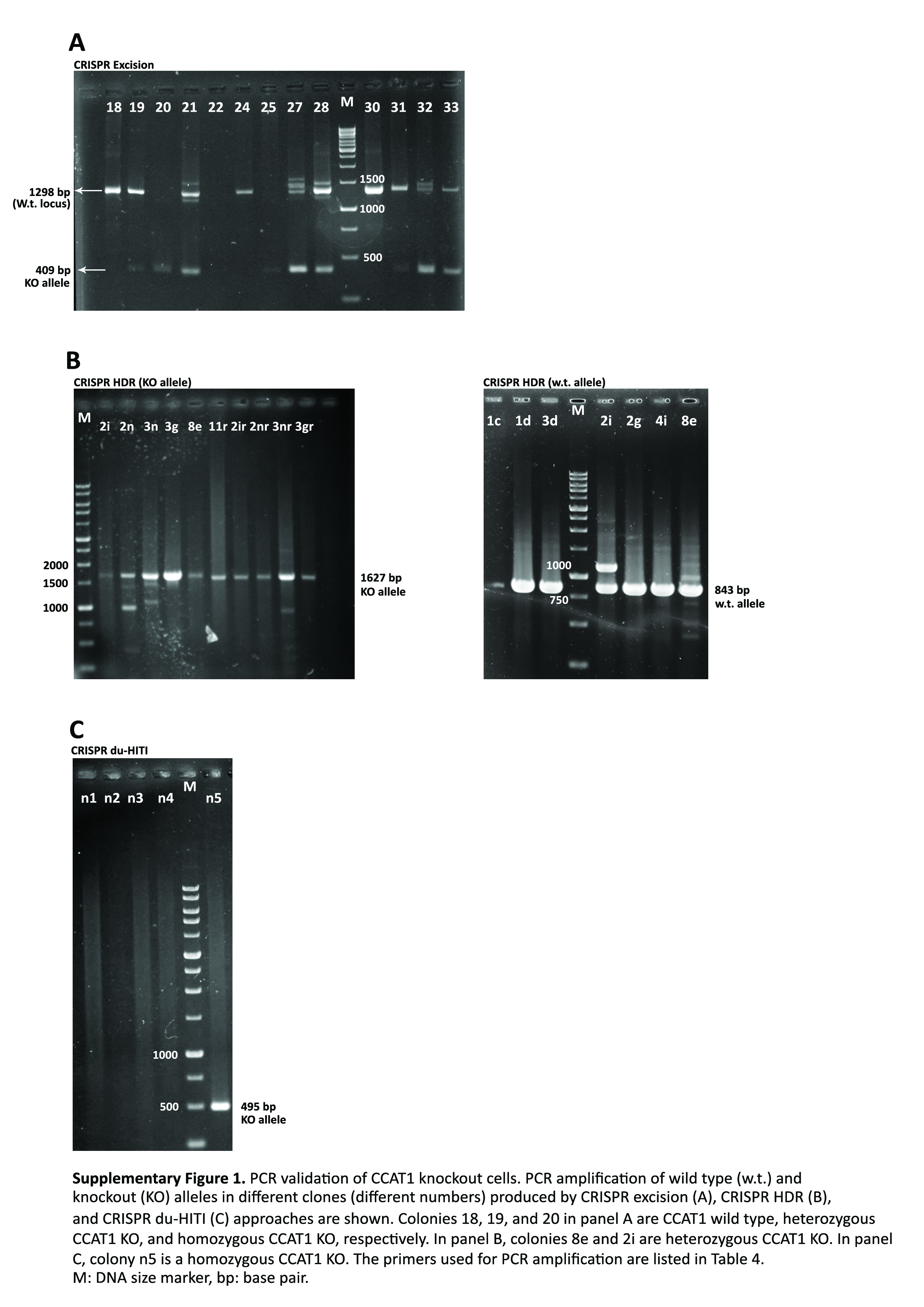

Supplement: Supplementary file 1 — Figure S1. PCR validation of CCAT1 knockout cells. PCR amplification of wild-type (w.t.) and knockout (KO) alleles in different clones (different numbers) produce by CRISPR excision (A), CRISPR HDR (B), and CRISPR du-HITI (C) approaches are shown. Colonies 18, 19, and 20 in panel A are CCAT1 wild-type, heterozygous CCAT1 KO, and homozygous CCAT1 KO, respectively. In panel B, colonies 8e and 2i are heterozygous CCAT1 KO. In panel C, colony n5 is a homozygous CCAT1 KO. The primers used for PCR amplification are listed in Table 4. M: DNA size marker, bp: base pair. (TIF 3887 kb) [file 12575_2018_86_MOESM1_ESM.tif]

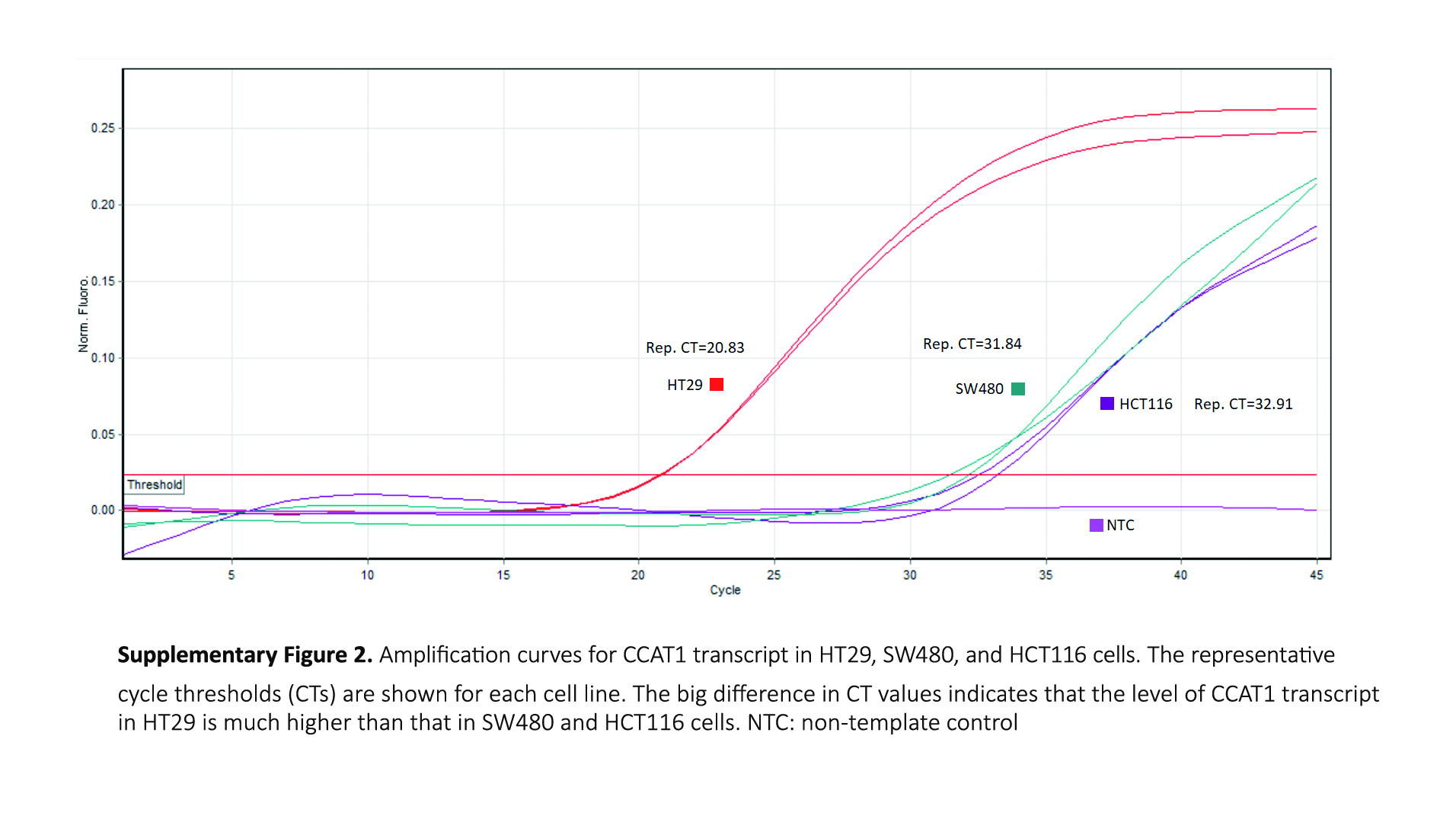

Supplement: Supplementary file 2 — Figure S2. Amplification curves for CCAT1 transcript in HT29, SW480, and HCT116 cells. The representative cycle thresholds (CTs) are shown for each cell line. The big difference in CT values indicates that the level of CCAT1 transcript in HT29 is much higher than that in SW480 and HCT116 cells. NTC: non-template control. (TIF 944 kb) [file 12575_2018_86_MOESM2_ESM.tif]
